# A Study on Methane and Nitrous Oxide Emissions Characteristics from Anthracite Circulating Fluidized Bed Power Plant in Korea

**DOI:** 10.1100/2012/468214

**Published:** 2012-04-30

**Authors:** Seehyung Lee, Jinsu Kim, Jeongwoo Lee, Eui-Chan Jeon

**Affiliations:** ^1^Department of Earth and Environmental Sciences, Sejong University, Seoul 143-747, Republic of Korea; ^2^Cooperate Course for Climate Change, Sejong University, Seoul 143-747, Republic of Korea

## Abstract

In order to tackle climate change effectively, the greenhouse gas emissions produced in Korea should be assessed precisely. To do so, the nation needs to accumulate country-specific data reflecting the specific circumstances surrounding Korea's emissions. This paper analyzed element contents of domestic anthracite, calorific value, and concentration of methane (CH_4_) and nitrous oxide (N_2_O) in the exhaust gases from circulating fluidized bed plant. The findings showed the concentration of CH_4_ and N_2_O in the flue gas to be 1.85 and 3.25 ppm, respectively, and emission factors were 0.486 and 2.198 kg/TJ, respectively. The CH_4_ emission factor in this paper was 52% lower than default emission factor presented by the IPCC. The N_2_O emission factor was estimated to be 46% higher than default emission factor presented by the IPCC. This discrepancy can be attributable to the different methods and conditions of combustion because the default emission factors suggested by IPCC take only fuel characteristics into consideration without combustion technologies. Therefore, Korea needs to facilitate research on a legion of fuel and energy consumption facilities to develop country-specific emission factors so that the nation can have a competitive edge in the international climate change convention in the years to come.

## 1. Introduction

The Kyoto Protocol, which was adopted in 1997 at the third Conference of Parties, specified the GHG reduction targets and action plans for Annex I countries. The Bali Road Map was agreed upon at the 13th session of the Conference of Parties, held in December 2007, which encourage developing nations with making voluntary efforts to reduce GHG emissions. Accordingly, Korea established “Comprehensive Plans on Combating Climate Change” (2008~2012), to capitalize on the crisis of climate change to create new opportunities to further advance our nation. The nation will be proactive in joining international efforts to reduce GHG emissions, and at the same time will take an early response to climate change at home to minimize the burden of GHG emission reduction [1].

Burden sharing, with regard to GHG emission reduction, includes technology transfer and GHG emission trade, as well as the reduction of the absolute quantity of emissions. In this context, it is vital to have precise information on the amount of emissions produced and the emission sources to seek methods of reducing GHG emissions. In other words, only if we can quantitatively estimate the spatial distribution and time variance of emissions in different emission sources can we establish a concrete strategy to reduce emissions. First and foremost, we must establish an inventory of emissions based on credible data in order to achieve the goal of mandatory emission reduction [2].

In this regard, it is essential for Korea to secure statistics on basic greenhouse gases that reflect the nation's circumstances and reality. GHG emissions will vary depending on intrinsic factors including fuel type, fuel property, different types of boiler and control facilities, and the amount of fuel consumption. In particular, CH_4_ and N_2_O emissions are influenced by numerous additional factors, some of which are hidden, such as conditions of consumption and operation as well as technical elements [3]. Accordingly, IPCC recommends that nations apply country-specific or technology-specific emission factors rather than default emission factors of IPCC when assessing national GHG emissions [3]. However, Korea has used the default emission factors due to the lack of relevant research within the nation. Therefore, it is fundamental to assess the emission factors befitting national circumstances in order to predict and estimate GHG emissions and establish emission reduction plans.

Although the anthracite coal-fired power plants in Korea consume a significant amount of domestic anthracite coal, there is virtually no research to assess emission factors on these power plants [4]. In particular, designated power plants in this study are one of the world's largest scaled anthracite coal-fired fluidized bed power plants [5].

Therefore, it should be categorized as a major GHG emission source in mapping out Korean GHG inventory as the plant uses anthracite produced in Korea and is exclusively a domestic technology. To this end, we need to develop Korean country-specific emission factors on fluidized power plants.

## 2. Selection of Facilities and Methods of Sample Extraction

### 2.1. Selection of Facilities

The research center designated first and second anthracite-fired fluidized bed power plants that use domestic low-grade anthracite. The details of the facilities are presented in Table 1. Each capacity of the facilities is 200 MW, respectively, and the total amount of power generation stood at approximately 2.2 TWh (as of 2005). An electric precipitator is installed as the first air pollution control facility, and a filter dust collector is installed as the second control facility.

The designated power plants are the only anthracite-fired power plants in Korea, which are run in the manner of circulating fluidized combustion, but not in the mixture combustion of pulverized coal and heavy oil. Annually, anthracite is consumed approximately 1.1 million tons, it means 25% of total domestic anthracite production, and 40% of total consumption from domestic anthracite-fired power plants. The combustion temperature of a fluidized bed is 900°C, significantly lower than pulverized coal combustion (1,200~1,500°C). The concentration of NO_*x*_ is 60 ppm, a figure much lower than 350 ppm, the effluent quality standard of  NO_*x*_.

### 2.2. Method of Sample Extraction

**Figure 1:**
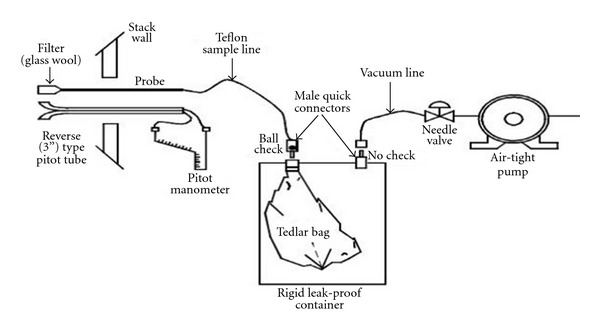
Diagram of greenhouse gas sampling system.

In this paper, EPA Method 18 (Figure 1) was applied when extracting GHG samples to assess the emission factor from GHG emitted from the plants [6]. A variety of tests were conducted to assess the temperature of flue gas, the amount of moisture, flow velocity, pressure, temperature, and others during sample extraction [7]. In order to reduce the margin of error, a one-liter Tedlar bag (SKC, US) was used for GHG sample extraction.

Fuel samples were extracted from the sample extractor installed at a coal conveyor, which provides coal from the coal center to a boiler when replacing fuel. The samples for moisture measurement were extracted through two stages on each level in a swift manner. The rest of the samples were reduced through quartering, and then final samples were extracted. The fuel extraction was conducted at each power plant, and the extracted anthracite was the fuel used as the exhaust gases were extracted.

## 3. Analysis Methods

### 3.1. Fuel Analysis Methods

#### 3.1.1. Element Contents Analysis Method

The contents of carbon and hydrogen are highly important in order to assess GHG emissions in the consumption of fossil fuel. It is essential to analyze elements of coal, because the fuel properties vary greatly depending on the origin. The same anthracite that is used for sample extraction in the research was analyzed by Thermo Finnigan-Flash EA 1112, USA, at a laboratory for analysis of Carbon, Nitrogen, Sulfur, Hydrogen, and other elements. A two-meter ParaQX column was used in the process. The reproducibility result of elemental analysis showed that the absolute difference of Carbon and Hydrogen was between 0.10%*∼*0.37% and between 0.15%*∼*0.25%, respectively, as shown in Table 2 when using BBOT (2,5-bis (5-tert-butyl-benzoxazolyl) thiophene) as samples. 

#### 3.1.2. Calorific Value Analysis Method

The calorific values of anthracite samples were analyzed with an automatic analyzer (IKA-C2000, Germany) at a laboratory. Injected samples were quantified from an electronic scale (Mettler Toledo-AB204S, Switzerland) with 0.1 mg sensitivity. The water temperature of the analyzer was set at 25°C using a temperature control device (KV-500, Germany) to analyze calorific values in Isoperibolic Mode. Cooling water used for the analyzer was created with a pure water apparatus (Duplex-150H, Korea). The reproducibility of anthracite was analyzed five times. The reproducibility was exceptional, with relative standard error (RSE) standing at 0.008%, as shown in Table 3.

### 3.2. Exhausted Gas Analysis Method

#### 3.2.1. Concentration of Methane and Nitrous Oxide Analysis Method

The concentration of CH_4_ and N_2_O was analyzed at a laboratory after extracting exhaust gases using a Tedlar bag. The analysis was conducted via gas chromatography (Model CP-3800, Varian, USA) in order to quantify the concentration of CH_4_ and N_2_O. Flame Ionization Detector (FID) and Electron Capture Detector (ECD) were connected to analyze CH_4_ and N_2_O. In this stage, one meter and three meters of Porapack QX 80/100 mesh columns (Stainless steel, external diameter of 3.175 mm, Restek) were used. The temperatures for the injector, oven, and detector were maintained at 120°C, 70°C, and 320°C, respectively. Ultrapure Nitrogen (99.9999%) was used as the carrier gas. Ten-port, six-port, and four-port gas switching valves were used when injecting samples in order to remove oxygen and moisture.

**Figure 2:**
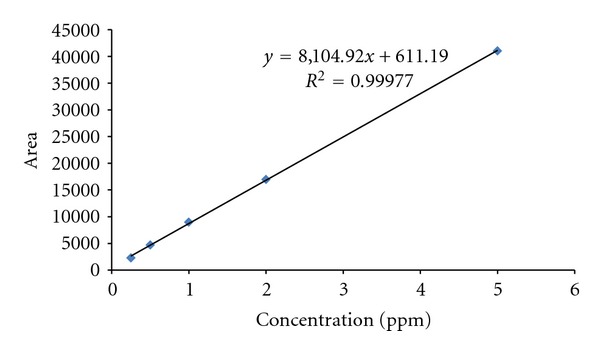
Result of calibration slope using CH_4_ standard gas.

**Figure 3:**
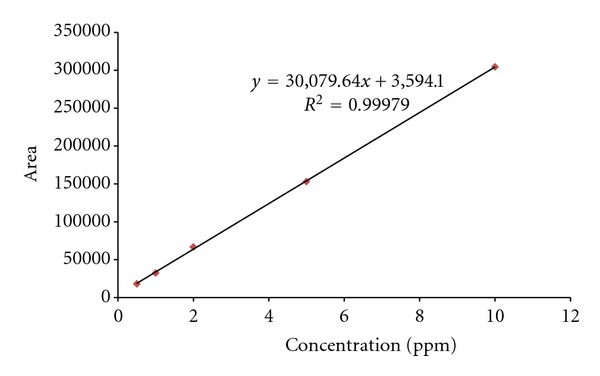
Result of calibration slope using N_2_O standard gas.

A calibration line was drawn up for each element before analysis in order to conduct a quantitative analysis for CH_4_ and N_2_O, and it was used for the concentration assessment. The calibration line was made based on five different samples with varied concentrations, ranging from 0.25–5 *μ*mol/mol for CH_4_. The calibration line for N_2_O was drawn from five different samples with varied concentrations ranging from 0.5~10 *μ*mol/mol. As a result, *R* ^2^ values of CH_4_ and N_2_O were 0.99977 and 0.99979, respectively, showing high relevance. Calibration results for CH_4_ and N_2_O were presented in Figures 2 and 3, respectively.

In order to confirm the reproducibility of CH_4_ analysis, standard gas (RIGAS, KOREA) with a concentration of 1.1 *μ*mol/mol was analyzed ten times repeatedly. A standard gas (RIGAS, KOREA) with a concentration of 1.0 *μ*mol/mol was analyzed ten times repetitively for reproducibility confirmation of N_2_O analysis. The result of reproducibility analysis was presented in Tables 4 and 5. The Relative Standard Error (RSE) of CH_4_ and N_2_O stood at 0.19340% and 0.57101%, respectively, displaying excellent reproducibility.

#### 3.2.2. Moisture Content of Exhaust Gas Analysis Method

Moisture in the exhaust gas emitted from the power plants was measured from a moisture sampling apparatus (M-5, Astek Korea) and an electronic scale (Ohaus adventurer, USA). Heat rays were installed inside the tubes of the moisture sampling apparatus so that moisture in the exhaust gas can condense inside the tubes, maintaining a temperature at 120°C to extract moisture. In order to measure the amount of moisture, a certain amount of granular anhydrous calcium chloride was filled into a round-shape absorption bottle as a moisture absorbent connected to sampling tubes for GHG. The gases were measured down to two decimal places (EPA method 4) on an integrated flow meter installed to a moisture sampling apparatus. After extracting the samples, the absorption bottle was weighed with a cap on it. The amount of moisture in the exhaust gases was measured, taking numerous factors into consideration including the differences of the bottle weight before and after extracting the samples, the flow rate of the samples, and the temperature of the gases.

#### 3.2.3. Estimated Method of Emission Factor

The elemental analysis of fuel enables a reliable assessment of emission factors for CO_2_. However, the emission factors for CH_4_ and N_2_O are susceptible to numerous variables of combustion conditions, such as combustion technology and its management. Therefore, it is hard to use the emission factors based on fuel analysis itself as a representative value [3]. Accordingly, emission factors for CH_4_ and N_2_O were assessed after precise measurement of the exhaust gas concentration from the power plants used in this research. There are four stages of work sheets for emission factor assessment through measurement. The measured concentration and flow rate of CH_4_ and N_2_O are inputted, and the unit is converted for the emission factor assessment in the first stage. The energy unit for fuel consumption is standardized in the second stage, and the amount of fuel consumption, power generation, and heat production are inputted in the third stage. In the fourth stage, the analysis for the calorific value of fuel is conducted, and the calorific value is inputted to assess CH_4_ and N_2_O emissions, and the same for CH_4_ and N_2_O emission factors in the fifth stage.

## 4. Results and Considerations

### 4.1. Analysis Results of Fuel Properties

The results from proximate analysis and elemental analysis for domestic anthracite used in the power plants are presented in Table 6. The carbon contents in the anthracite on dry basis stood at 65.35%, while hydrogen stood at 1.46%. The proximate analysis of volatile components, fixed carbon, and inherent moisture stood at 7.16%, 57.94%, and 4.42%, respectively. When comparing the results of proximate analysis and elemental analysis with the results of a previous anthracite analysis from different anthracite-fired plants, the carbon contents were similar at 62.26%. The hydrogen contents were nearly identical at 1.12% [8].

**Figure 4:**
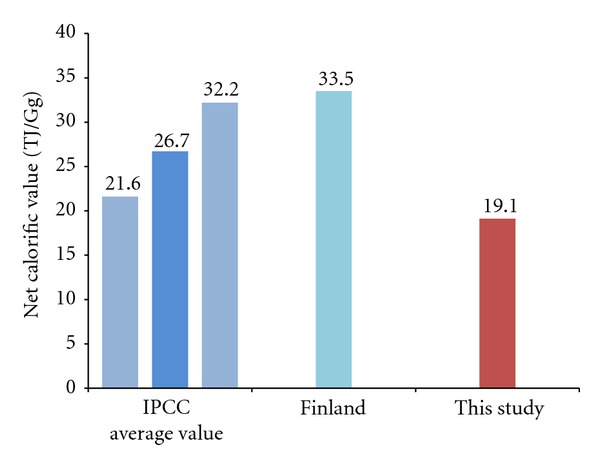
Comparison of net calorific value by this study and the IPCC.

Measuring calorific values, the calorific values as received basis with the application of total moisture displayed between 4,723–4,779 kcal/kg. The measured calorific values were compared with the standard calorific values of anthracite presented by the IPCC, as shown in Figure 4. Low calorific values of anthracite presented by IPCC G/L in 2006 are 26.7 TJ/Gg, and the margin of error within the 95% confidence interval is between 21.6~32.2 TJ/Gg. On the other hand, when the unit used in calculating calorific values of domestic anthracite from research-designated power plants is converted in order to compare with the values presented by the IPCC, the figure was found to be as low as at 19.1 TJ/Gg. The disparities of calorific values in different countries are attributable to the variances in the composition and origins of coal. These variables are decisive factors in determining the GHG emission factors; therefore, we need to develop a country-specific emission factors.

### 4.2. Analysis Results of Methane and Nitrous Oxide Emissions

The numerous analysis results, which include the concentration of CH_4_ and N_2_O emissions, the temperature of exhaust gas, and the moisture and emissions measured from research-designated power plants, are shown in Table 7. The average CH_4_ concentration from the first exhaust pipe of the power plant was measured at 2.22 ppm, and the second exhaust pipe at 1.41 ppm. The average concentration of total emissions stood at 1.85 ppm. The average N_2_O concentrations from each exhaust pipe were measured at a value between 2.78~3.72 ppm, and the average concentration of emissions from all facilities stood at 3.25 ppm. The research found that there were slight differences in the concentrations of CH_4_ and N_2_O emissions from some exhaust pipes, even though they used the same energy source. The disparities are likely attested to the concentrations of CH_4_ and N_2_O, which are more susceptible to circumstantial factors such as combustion conditions, fuel amount, and flow rate.

### 4.3. Estimated Results of Emission Factors of Methane and Nitrous Oxide Emissions

In order to assess the CH_4_ and N_2_O emission factors of fluidized bed power plants that consume anthracite as their energy source, the low calorific values were calculated through fuel analysis. CH_4_ and N_2_O emission factors were assessed using the element's concentration out of exhaust gases extracted from exhaust pipes, the emission flow rate of Tele-monitoring system (TMS), and the amount of power generation. The assessed emission factors are presented in Table 8. The emission factors for CH_4_ assessed from the anthracite-fired fluidized bed power plants in this research stood at 0.486 kg/TJ, 50% lower than the default emission factors for anthracite presented by the IPCC. Still, the assessed emission factors fell into the range of emission factors for CH_4_ presented by the IPCC.

Emission factors for N_2_O were assessed at 2.198 kg/TJ, 30% higher than the default emission factors for anthracite presented by the IPCC. The assessed emission factors for N_2_O fell into the range of emission factors presented by the IPCC. The emission factors for CH_4_ are 1 kg/TJ at circulating fluidized bed power plants with less than 5 MW facility capacity and 4 kg/TJ at plants with more than 5 MW. It shows that CH_4_ and N_2_O emissions can vary from power plants with the same method for combustion. The emission factors for N_2_O presented by Finland are 30 kg/TJ, which is particularly distinctive from this research or the emission factors presented by the IPCC. Likewise, emission characteristics for CH_4_ and N_2_O can vary depending on different conditions, such as the country of origin or the method for combustion.

## 5. Conclusion

We selected circulating fluidized bed power plants as research facilities, which consume 25% of domestically produced anthracite. In order to analyze GHG emission factors of anthracite-fired circulating fluidized bed power plants, we assessed the calorific values and carbon contents for domestic low-grade anthracite through fuel analysis for domestic coal. We conducted an analysis on CH_4_ and N_2_O emission concentration from the stack of the plants. With the analysis results, we assessed GHG emission factors for CH_4_ and N_2_O.

The fuel analysis showed that the low calorific value for coal used at the research-designated facility was at 19.1 TJ/Gg. The analysis of CH_4_ and N_2_O concentration from exhausted gas showed that the average emission concentrations for CH_4_ and N_2_O from research-designated facilities were at 1.82 ppm and 3.25 ppm, respectively. The emission factors for CH_4_ and N_2_O from the same analysis stood at 0.486 kg/TJ and 2.198 kg/TJ, respectively. The assessed emission factors for CH_4_ from anthracite-fired circulating fluidized bed power plants were 52% lower than 1 kg/TJ, the default emission factors for anthracite presented by the IPCC. The disparities were due to differences in the method for and the conditions of combustion. The emission factors for N_2_O were assessed 46% higher than 1.5 kg/TJ, the emission factors presented by the IPCC. This disparity is attributable to the variance in method for combustion and other conditions because the default emission factors suggested by IPCC do not take combustion technologies into consideration. When calculate the national GHG emissions from these power plants using the emission factors in this study, CH_4_ emissions could be 52% lower than emissions calculated by IPCC emission factor. But N_2_O emissions should be 46% higher than emissions estimated used by emission factor of IPCC.

Therefore, it is vital to proactively promote research for developing country-specific emission factors on a wide variety of fuel and energy consumption facilities in order to secure a dominant position in future international negotiation on climate change convention.

## Figures and Tables

**Table 1:** Status of power generation of anthracite-fired fluidized bed plants and air-pollution control facilities (2005. 1.1.~12.31.).

Stack	Electric capacity (MW)	Electric generation				Control device	
		Gross generation (MWh)	Load factor (%)	Net generation (MWh)	Plant factor (%)	1st	2nd
1	200	1,149,264	63.7	1,027,830	65.42	ESP	Filter house
2	200	1,074,672	58.4	955,105	61.17	ESP	Filter house

Total	400	2,223,936		1,982,935			

Source: KEPCO(2006. 5.).

**Table 2:** Repeatability of elemental analysis for carbon (C) and hydrogen (H) in coal.

Times	Sample type	Injection weight (mg)	Element content (%)	
			C	H
1	BBOT	1.686	72.53	6.09
	Unknown^1^	1.832	72.90	6.04
	Absolute difference (%)		0.37	0.15

2	BBOT	1.686	72.53	6.09
	Unknown^1^	1.832	72.63	5.84
	Absolute difference (%)		0.10	0.25

^1^Unknown is that using the same BBOT sample but not inputting the information of the sulfanilamide element content.

**Table 3:** Repeatability test of calorific analysis using benzoic acid.

Times	Mass of benzoic acid (g)	Calorific value (kcal/kg)
1	0.9998	6,316
2	0.9994	6,315
3	1.0002	6,317
4	0.9993	6,315
5	0.9989	6,314

	Mean	6,315
	S.D.	1.14
	RSD (%)	0.018
	S.E.	0.5

**Table 4:** Repeatability test of concentration analysis using CH_4_ standard gas.

Times	Concentration of CH_4_ (*μ*mol/mol)
1	1.11226
2	1.10572
3	1.10449
4	1.10930
5	1.09610
6	1.09919
7	1.09141
8	1.09956
9	1.09746
10	1.09437

Mean	1.10099
S.D.	0.00673
RSD (%)	0.61160
S.E.	0.00213
RSE (%)	0.19340

**Table 5:** Repeatability test of concentration analysis using N_2_O standard gas.

Times	Concentration of N_2_O (*μ*mol/mol)
1	1.03801
2	1.01238
3	1.01570
4	0.98851
5	1.01171
6	0.98571
7	0.98551
8	1.00819
9	0.98588
10	0.98644

Mean	1.00180
S.D.	0.01809
RSD (%)	1.80570
S.E.	0.00572
RSE (%)	0.57101

**Table 6:** Results of proximate and elemental analysis of anthracite sampled at the power plants.

	Proximate analysis (air-dried basis), %				Elemental analysis (dry basis), %	
	IM	Ash	VM	FC	C	H
Stack 1	4.42	30.48	7.16	57.94	65.35	1.46
Stack 2	4.42	30.47	7.17	57.94	65.35	1.46

IM: Inherent moisture, VM: Volatile matter, FC: Fixed carbon, HHV: Higher heating value.

**Table 7:** Non-CO_2_ concentration and exit condition of exhaust gas from stacks in the anthracite Fluidized Bed power plants.

Stack no.	CH_4_ concentration (ppm)	N_2_O concentration (ppm)	Temperature(°C)		Moisture (g/m^3^)	Flow rate (m^3^/hr)
			Stack	Ambient		
1	2.22	3.72	151.40	32.00	63.8	566,898
2	1.41	2.78	151.00	32.00	63.8	626,667

**Table 8:** Non-CO_2_ emission factors of anthracite fluidized Bed power plant investigated in this study.

	CH_4_ emission factor (kg/TJ)	N_2_O emission factor (kg/TJ)
This study	0.486	2.198
IPCC (2006)^1^	1 (0.3~3)	1.5 (0.5~5)
FINLAND (2007)^2^	4 (>5 MW)	30
	1 (<5 MW)	30

^1^Non-CO_2_ default emission factor for anthracite.

^2^Non-CO_2_ emission factor for coal-fired circulating fluidized bed power plants.
